# Cognitive Performance and Long-term Exposure to Outdoor Air Pollution: Findings From the Harmonized Cognitive Assessment Protocol Substudy of the English Longitudinal Study of Ageing (ELSA-HCAP)

**DOI:** 10.1093/gerona/glaf060

**Published:** 2025-03-17

**Authors:** Giorgio Di Gessa, Mikaela Bloomberg, Rina So, Shaun Scholes, Thomas Byrne, Jinkook Lee, Sara D Adar, Paola Zaninotto

**Affiliations:** Department of Epidemiology and Public Health, University College London, London, UK; Department of Epidemiology and Public Health, University College London, London, UK; Department of Epidemiology and Public Health, University College London, London, UK; Department of Epidemiology and Public Health, University College London, London, UK; Department of Epidemiology and Public Health, University College London, London, UK; Department of Economics and Center for Economic and Social Research, University of Southern California, Los Angeles, CA, USA; School of Public Health, University of Michigan, Ann Arbor, MI, USA; Department of Epidemiology and Public Health, University College London, London, UK; (Medical Sciences Section)

**Keywords:** Cognition, Emission sources, NO_2_, PM_2.5_, Pollution

## Abstract

**Background:**

Although air pollution is associated with worse cognitive performance, whether these relationships differ by cognitive domain and which sources of air pollution are particularly detrimental to cognition remains understudied. This study examined associations between cognitive scores across 3 domains in older adults and 8–10 years of exposure to air pollutants (NO_2_, total PM_2.5_, and PM_2.5_ from different emission sources).

**Methods:**

We used data from the 2018 Harmonized Cognitive Assessment Protocol substudy of the English Longitudinal Study of Ageing (*N* = 1 127). Outdoor concentrations of each pollutant were estimated for 2008/2010–2017 and summarized using means and group-based trajectories. Linear regression models were used to assess long-term air pollution exposure relationships with memory, executive function, language, and global cognitive function after adjustment for key individual and neighborhood-level confounders.

**Results:**

Associations between air pollution trajectories and cognition are mostly inverted j-shaped, with respondents exposed to the highest residential levels of NO_2_ and total PM_2.5_ having worse performance for global cognition (β = −.241; 95% CI = [−0.46, −0.02] and β = −.334; 95% CI = [−0.55, −0.12], respectively) than those exposed to average levels of pollution. Similar associations were also found for executive function and memory (PM_2.5_ only), whereas more compelling dose–response evidence was found for language. Higher emissions from industry and residential combustion, as well as biofuel, coal, and oil and natural gas combustion, were associated with worse language scores.

**Conclusions:**

Air pollution and its sources have domain-specific associations with cognitive performance, with most consistent evidence observed for language. Continued efforts to reduce air pollution, particularly where levels are the highest, might benefit cognitive performance.

Aging-related decline in cognitive function contributes to reductions in life expectancy, quality of life, and social participation (1–5). With a rapidly aging global population, identifying drivers of heterogeneity in cognitive function during aging is therefore a pressing public health issue. Among the modifiable factors associated with cognitive health, a growing body of evidence supports the link between exposure to outdoor air pollutants and worse cognitive function (6–10). In their report, Livingston et al. estimate the population-attributed fraction for air pollution for dementia at 2.6%, higher than the one calculated for hypertension and physical inactivity (11). In particular, exposure to nitrogen dioxide (NO_2_) and particulate matter with aerodynamic diameters less than 2.5 µm (PM_2.5_) are most implicated for cognitive health (8,9,12). Although the biological pathways underlying associations between air pollution and cognitive function are not yet fully understood, and specific air pollutants may affect the brain and cognitive health differently, it is hypothesized that air pollution adversely affects both the central nervous system and the circulatory system, leading to increased cognitive decline and risk of dementia (13,14). It is also understood that there may be direct impacts on the brain since the smallest particles can travel to the brain through the olfactory bulb and cross the blood–brain barrier (15).

Several knowledge gaps remain in this area of research. First, few studies have investigated the links between air pollution and specific cognitive domains, with findings inconsistent in direction and magnitude depending on the type of pollutant and the cognitive domain considered (16–23). For instance, Gatto et al. (19) reported no association between exposure to NO_2_ and executive function but found increasing exposure to PM_2.5_ associated with lower verbal learning among healthy residents living in the Los Angeles, California area of the United States. Tonne et al. (20), however, found that increased exposure to PM_2.5_ was associated with reduced reasoning ability among Whitehall II study residents of Greater London, United Kingdom. Using the UK Biobank, Cullen et al. (21) found that exposure to NO_2_ was associated with better reasoning scores but lower visuospatial memory. Second, PM_2.5_ originates from many sources in the environment, including traffic, coal-fired power plants, and agricultural emissions, and each source can emit PM_2.5_ with distinct physical and chemical characteristics. For example, components such as black carbon (BC) and nitrates are more common in PM_2.5_ from traffic-related sources, whereas ammonium is often in PM_2.5_ from agriculture. However, to date, few studies have examined associations between specific constituents of PM_2.5_ and cognitive function, with most focusing on BC and traffic-related exposures (23–26). The only study so far that has considered broader sources of PM_2.5_ emissions has focused on dementia and found that PM_2.5_ from agriculture and wildfires were particularly detrimental for incident dementia in the United States (27). Finally, although it is common practice to examine the association between air pollutants and cognitive function by taking the mean concentration of air pollutants over the study period, this approach might not account for different patterns and levels of exposure to air pollutants over time.

In this study, we attempt to fill these gaps in knowledge by examining associations of cognitive function with long-term exposure to NO_2_, total PM_2.5_, and source-specific PM_2.5_ measured over a period of up to 10 years. Cognitive function is assessed using the Harmonized Cognitive Assessment Protocol (HCAP), a detailed set of neuropsychological assessments designed to measure key cognitive domains affected by cognitive aging (including memory, executive function, and language) and facilitate cross-national comparisons between national cohort studies of aging. We examine long-term exposure to air pollution using both mean air pollution concentration during the study period as well as by classifying participants into 10-year trajectories of air pollution concentrations.

## Method

### Study Population

Our study population was adults aged 65 years and older living in private households in England in 2018. We used data from Wave 4 (2008–2009) of the English Longitudinal Study of Ageing (ELSA) and Wave 1 (2018) of the HCAP substudy of ELSA (ELSA-HCAP). ELSA is a nationally representative cohort study of adults aged 50 years and older, living in private households in England (28). ELSA started in 2002, and data are collected biennially using face-to-face personal interviews and self-completion questionnaires; more than 18 000 people have taken part in the study since its inception. Refreshment samples of new participants have been recruited regularly to maintain the age profile and ensure that the study remains representative of the English population aged 50 years and older.

The HCAP was implemented in a subset of ELSA participants to examine mild cognitive impairment and dementia using more detailed assessments of cognitive function than possible in the main interviews of ELSA (29). A probability sample of ELSA participants who did not use a proxy for their core ELSA interview, were aged 65 years or older in January 2018, and had completed an ELSA interview in person at either Wave 8 (2016–2017) or Wave 7 (2014–2015) were invited to participate in ELSA-HCAP interviews that took place between January and April 2018. To ensure adequate sample sizes of participants with dementia, participants with low cognitive scores (assessed using telephone interview cognitive screening and/or Alzheimer’s disease or dementia previously self-reported in ELSA interviews) were oversampled. Of the 1 684 eligible respondents invited to the study, 1 272 completed the face-to-face HCAP interview (response rate 76%). More details of ELSA and ELSA-HCAP surveys’ sampling frame, methodology, and questionnaires can be found at www.elsa-project.ac.uk. ELSA was approved by the London Multicentre Research Ethics Committee (MREC/01/2/91), and the ELSA-HCAP substudy received ethical approval from the South Central-Berkshire National Health Services (NHS) Research Ethics Committee. Informed consent was obtained from all participants or their guardians. All ELSA data are available through the UK Data Service (SN 5050 and 8502).

In the present study, demographic and socioeconomic characteristics of ELSA-HCAP participants were drawn from ELSA core interviews. Though most ELSA-HCAP participants first participated in ELSA during Wave 1 (2002), 257 (20%) were first interviewed in Wave 4 (2008–2009); to retain these individuals, we therefore considered ELSA Wave 4 (2008–2009) as the baseline wave in the analyses.

### Exposure Assessment

Environmental exposome data are drawn from the Gateway to Global Aging Data (https://exposome.g2aging.org). Full details on the environmental exposome data, their spatial and temporal resolutions, and links to the original source information are presented by D’Souza et al. (30) and can be found at the Gateway Exposome site. Briefly, spatiotemporal prediction models were used to estimate annual average estimates of total NO_2_ concentrations and PM_2.5_ levels for each ELSA-HCAP participant. All air pollution measures were estimated using the point location of each respondent’s exact residential address. Estimates for NO_2_ were predicted at a resolution of 50 m × 50 m from 2005 to 2019 and were generated using models that included remote sensing from the Ozone Monitoring Instrument, road networks, built environments, and meteorological variables (31). Annual mean concentrations of total PM_2.5_ were available at a 1-km^2^ resolution from 2010 to 2019 and were created using the Data Integration Model for Air Quality that combines information from satellite remote sensing and chemical transport models, meteorological information, correlations over space, and ground-based monitoring data (32). As ELSA Wave 4 (2008–2009) was the baseline wave for this study, we considered air pollution data recorded between 2008 and 2017 for NO_2_ and between 2010 and 2017 for PM_2.5_.

Source-specific PM_2.5_ concentrations were derived by multiplying the yearly average PM_2.5_ concentration at each address by local fractions of PM_2.5_ attributable to different emission sources. These fractions were generated at a resolution of 0.5° × 0.625° (~55 km × ~70 km) by serially running an atmospheric chemistry-transport model (GEOS-Chem) with all sources but one to isolate the unique contribution of that source to the total PM_2.5_ mixture (33). Although these emission-specific fractions were generated using data from 2017, prior evidence suggests that these estimates are representative for previous years as well (27). In this study, we focused on agriculture, energy production, industry, residential combustion, and road traffic as sector-specific sources of PM_2.5_. Altogether, these sources accounted for ~61% of the total PM_2.5_ emissions. We also considered fuel-specific sources with primary emissions including solid biofuel, coal, and liquid oil and natural gas combustion. These sources and the percentage of total PM_2.5_ they account for are described in Supplementary Table S1.

### Cognitive Function

The HCAP battery was designed to assess key cognitive domains affected by cognitive aging, including memory, executive function, and language. In ELSA-HCAP, respondents were administered a range of cognitive tests adapted from the original battery by Langa et al. (34); these included well-established neurocognitive assessments such as the “East Boston Memory Test” and the “Wechsler Memory Scale,” immediate and delayed recall, backwards counting tasks, and shape drawing. Details of cognitive testing protocols and tests used (with relevant references) are available in the ELSA-HCAP Technical Report (35) and its profile description (29). Cognitive scores for each cognitive domain were produced using the procedure described by Gross et al. (36) and were derived using factor analysis of multiple cognitive tests relevant to each domain. These scores have been shown to be highly reliable and valuable for population-based research on cognition. The derived cognitive scores were normally distributed (Supplementary Figure S1); to facilitate their interpretation, scores were standardized using the mean and standard deviation of the analytic sample, with positive scores representing above-the-average scores.

### Covariates

Potential confounders of the relationship between long-term exposure to outdoor air pollution and cognitive function were drawn from ELSA Wave 4 (2008–2009) and included age, sex, age at which participants completed their highest education qualification, and total wealth, defined as the sum of financial, physical, and housing wealth (divided into quintiles). We also included a summary measure of cognitive function (based on tests of immediate and delayed recall and verbal fluency administered in ELSA Wave 4). Based on each participant’s residential location, we also included urbanicity, measured according to the 2001 Census Urban/Rural Indicator, and neighborhood socioeconomic status (in quintiles) measured using the 2007 Index of Multiple Deprivation that accounts for area dimensions including income, employment, living environment, and crime (37). We considered a minimum set of covariates that would not fall in the causal pathway between long-term exposure to air pollution and cognition, as these mediators could underestimate the effect of long-term air pollution on cognition or introduce bias in our results. Therefore, we did not control for health conditions (such as cardiovascular, respiratory, or other chronic diseases) or health behaviors (such as smoking or physical activity) (Supplementary Figure S2).

### Statistical Analysis

We adopted 2 strategies to summarize long-term exposure to air pollution. First, for each of the air pollutants (NO_2_, total PM_2.5_, and source-specific PM_2.5_), we calculated mean-centered average concentrations and interquartile range (IQR) over the period under study (2008–2017 for NO_2_; 2010–2017 for PM_2.5_), in line with previous literature on long-term exposure to outdoor air pollution. Second, we applied group-based trajectory modeling (38) to investigate if there were distinctive trajectory patterns of exposure to different air pollutants over time and understand how these trajectories relate to cognitive function.

A group-based trajectory modeling framework takes into account the dependency of observations and assumes a mixture of subpopulations with different individual trajectories within the target population and identifies distinctive groups within which individuals share similar trajectories (39,40). Both linear and nonlinear trajectories can be captured by introducing higher-order polynomial growth parameters into the model. For each subject, the model provides the probability of belonging to each of the identified trajectory groups and assigns the subject to the trajectory group based on the highest probability. To determine the optimal number of trajectory groups within our sample, we fitted unconditional group-based trajectory models using up to 10 years of data (2008–2017) on outdoor air pollution, with missing data for air pollutants handled using full information maximum likelihood estimation. We tested 1–7 trajectory groups, with the optimal number of groups selected using a wide range of criteria including the Akaike Information Criterion (AIC), the Bayesian Information Criterion (BIC), and its sample size-corrected version (c-BIC). For each of these, lower scores indicate (relatively) better fitting models. We also considered the following criteria: overall average posterior probabilities of group membership as a measure of classification quality (APPA is an entropy index, with values approaching 1.0 indicating a favorable classification); group size (no trajectory groups should include <5% of participants to ensure reproducibility of the results); the usefulness of the number of groups in terms of the similarities/differences in their trajectory shapes; and the interpretability of these distinctive trajectories (38,39). After determining the optimal number of trajectory groups, we established the optimal shape of the trajectory by testing growth parameters for each trajectory group up to the fifth degree. Higher-order growth parameters (quadratic to quintic) were dropped if not statistically significant (*p* < .05). Trajectories were estimated for NO_2_, PM_2.5_, and each of the source-specific PM_2.5_ concentrations.

We used linear regression models to estimate associations between air pollution and cognitive performance for NO_2_ and total PM_2.5_, then for source-specific PM_2.5_. These models first included mean air pollution during the study period (2008–2017 for NO_2_; 2010–2017 for PM_2.5_) fitted as continuous exposures. We then fitted models including the air pollution trajectory group as categorical exposure; we chose as the reference trajectory group the one with the trajectory closest to the mean concentration of that pollutant during the study period (as this level was used for the continuous exposures). For both continuous and categorical exposures, we fitted basic models (Model 1), where we adjusted for age and sex, and fully adjusted models (Model 2), where we further adjusted for education, wealth, urbanicity, area-level deprivation, and the summary cognitive function measure assessed at baseline. All models were weighted to account for differential probability of selection into ELSA-HCAP, nonresponse (especially for the low cognition group, which had the lowest response rate), and the ELSA study design (35). Data management, trajectories, and statistical analyses were performed using Stata/MP 18.0 (and the *traj* plugin) (41,42).

## Results

### Sample Descriptives; NO_2_ and PM_2.5_ Pollution Mean and Trajectories

For this study, we selected all HCAP-ELSA respondents with no missing data on the exposure, outcome, or key confounders described above, with a final analytical sample of 1 172 respondents. At ELSA Wave 4, respondents were aged 65 years on average (*SD* = 7); 54% were female, and 77% were living in an urban area, with 25% in the highest wealth quintile and 14% living in the most deprived area quintile (Table 1). The mean (*SD*) 10-year average NO_2_ concentration between 2008 and 2017 was 22.89 (6.36) μg/m^3^ and it was 11.89 (1.53) μg/m^3^ for total PM_2.5_ between 2010 and 2017.

**Table 1.** Characteristics of the Study Population

ELSA Characteristics	
Mean age at baseline in years (*SD*)	65.37 (7.13)
Sex	
Male	46.3% (*N* = 533)
Female	53.7% (*N* = 639)
Wealth quintiles	
Lowest wealth	16.3% (*N* = 186)
Second	18.5% (*N* = 232)
Middle	22.2% (*N* = 261)
Fourth	18.5% (*N* = 222)
Highest wealth	24.5% (*N* = 271)
Age at completion of highest education qualification	
14 or younger	12.7% (*N* = 177)
15	40.5% (*N* = 474)
16	19.6% (*N* = 228)
17	8.1% (*N* = 81)
18	4.7% (*N* = 57)
19 or older	14.4% (*N* = 155)
Urbanicity	
Urban	77.3% (*N* = 880)
Rural	22.7% (*N* = 292)
Quintile index of multiple deprivation score	
0.37–8.32 (least deprived)	21.7% (*N* = 268)
8.32–13.74	25.2% (*N* = 295)
13.74–21.22	20.6% (*N* = 248)
21.22–34.42	18.4% (*N* = 209)
34.42–85.46 (most deprived)	14.1% (*N* = 152)
Cognitive function (range of scores)	
Overall cognition	−2.84; 2.42
Executive function	−2.77; 2.30
Language	−2.12; 3.77
Memory	−2.81; 1.89
Mean air pollutants pre-HCAP interview (*SD*)	
Nitrogen dioxide (NO_2_)	22.89 (*SD* = 6.36)
Total fine particulate matter (PM_2.5_), μg/m^3^	11.89 (*SD* = 1.53)
Agriculture	3.21 (*SD* = 0.46)
Energy	1.07 (*SD* = 0.26)
Industry	0.96 (*SD* = 0.21)
Residential	1.04 (*SD* = 0.26)
Road traffic	1.05 (*SD* = 0.17)
Biofuel	1.24 (*SD* = 0.33)
Coal	0.65 (*SD* = 0.14)
Oil and gas	3.18 (*SD* = 0.43)

*Notes*: Sources: English Longitudinal Study of Ageing (ELSA) and Gateway to Global Aging Environmental Exposome Data for England. The sample is restricted to participants of the Harmonized Cognitive Assessment Protocol (HCAP) substudy of ELSA (*N* = 1 127). Weighted ELSA data. Data are reported as percentages (numbers) unless otherwise indicated.

**Figure 1.**
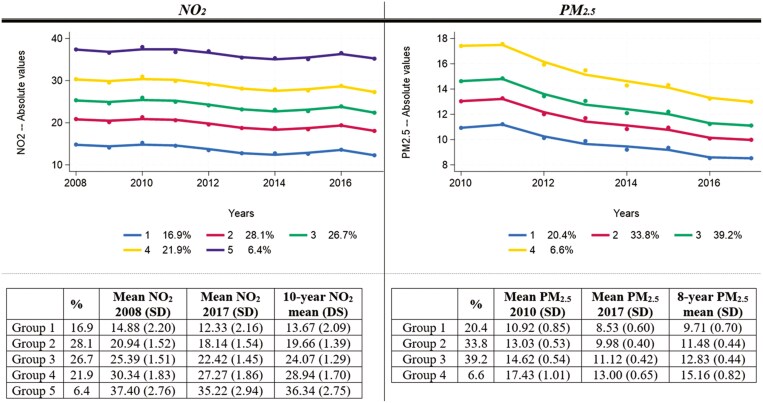
Trajectories of outdoor air pollution and characteristics of the groups. Sources: Gateway to Global Aging Environmental Exposome Data for England. The sample is restricted to participants of the Harmonized Cognitive Assessment Protocol substudy of the English Longitudinal Study of Ageing (ELSA-HCAP, *N* = 1 127). NO_2_ (µg/m^3^) = nitrogen dioxide; PM_2.5_ (µg/m^3^) = particulate matter with aerodynamic diameters less than 2.5 μm. Best-fitting trajectory groups were obtained using group-based trajectory modeling—see Supplementary Tables S3 and S4 for details.

Over the years under study, there was a general decline in both levels of NO_2_ and PM_2.5_, with the mean levels of NO_2_ reducing from 24.18 μg/m^3^ in 2008 to 21.36 μg/m^3^ in 2017, and for PM_2.5_ from 13.52 μg/m^3^ in 2010 to 10.33 μg/m^3^ in 2017 (Figure 1, Supplementary Table S2). As shown in Figure 1, we identified 5 NO_2_ exposure trajectories (Supplementary Table S3) and 4 PM_2.5_ trajectories (Supplementary Table S4). Although the slopes of the trajectory groups were statistically different and the changes in absolute values of air pollutants over time were not equal across groups, substantially the trajectory groups were largely parallel and are labeled in descending order of average exposure (groups 1–5 for NO_2_ and groups 1–4 for PM_2.5_). For instance, 20% of the respondents were exposed to a mean level of 9.71 μg/m^3^ of PM_2.5_ (with values reducing by 2.38 μg/m^3^ from 10.92 in 2010 to 8.53 μg/m^3^ in 2017) compared to 7% of the sample who were exposed to higher levels of PM_2.5_ (an average of 15.16 μg/m^3^, reducing by 4.43 μg/m^3^ from 17.43 μg/m^3^ in 2010 to 13.00 μg/m^3^ in 2017). Source-specific and fuel-specific PM_2.5_ values are presented in Table 1, and their trajectories are presented in Supplementary Figures S3 and S4.

### Outdoor Air Pollution and Cognitive Performance

Table 2 presents the associations between cognitive performance and NO_2_ and total PM_2.5_ for each cognitive domain. When we considered air pollution as a continuous exposure, in models only adjusted for age and sex (Model 1), higher NO_2_ concentrations were generally associated with lower overall cognitive performance (β = −.013; 95% CI = [−0.02, −0.00]), executive function (β = −.016; 95% CI = [−0.03, −0.01]), and language (β = −.016; 95% CI = [−0.03, −0.01]). However, adjustment for other covariates weakened these associations and the main model findings (Model 2) remained statistically significant only for language (β = −.013; 95% CI = [−0.02, −0.00]). When we considered groups of exposure to NO_2_, results suggest that even accounting for key personal and neighborhood confounders, compared to those who experienced an average level of NO_2_ between 2008 and 2017 of 24.07 μg/m^3^, respondents in the group of highest exposure (at an average level of 36.34 μg/m^3^) had worse performance for overall cognitive function (β = −.241; 95% CI = [−0.46, −0.02]), executive function (β = −.291; 95% CI = [−0.54, −0.04]), and language [β = −.328; 95% CI = [−0.59, −0.07]). Regardless of whether NO_2_ exposure was considered as a continuous or categorical variable, no associations between memory and NO_2_ were found although the direction of association generally suggested lower scores of cognitive performance on average with higher exposure.

**Table 2.** Associations (95% confidence intervals) Between Outdoor Air Pollution Concentrations (NO_2_ and total PM_2.5_) and Cognitive Performance in the ELSA-HCAP (2018)

	Overall Cognition		Executive Function		Language		Memory	
NO_2_	Model 1	Model 2	Model 1	Model 2	Model 1	Model 2	Model 1	Model 2
Mean NO_2_ (centered)	−0.013** (−0.02, −0.00)	−0.007 (−0.02, 0.00)	−0.016** (−0.03, −0.01)	−0.010 (−0.02, 0.00)	−0.016** (−0.03, −0.01)	−0.013* (−0.02, −0.00)	−0.005 (−0.01, 0.00)	−0.002 (−0.01, 0.01)
Group 1 (*mean 13.7 μg/m*^*3*^)	−0.006 (−0.18, 0.16)	−0.117 (−0.28, 0.05)	−0.017 (−0.19, 0.16)	−0.152 (−0.32, 0.02)	0.005 (−0.19, 0.20)	−0.044 (−0.25, 0.16)	−0.001 (−0.17, 0.17)	−0.069 (−0.25, 0.11)
Group 2 (*mean 19.7 μg/m*^*3*^)	0.062 (−0.07, 0.20)	−0.002 (−0.12, 0.11)	0.052 (−0.09, 0.19)	−0.006 (−0.12, 0.11)	0.082 (−0.08, 0.24)	0.030 (−0.12, 0.18)	0.039 (−0.10, 0.17)	−0.013 (−0.14, 0.11)
Group 3 (*mean 24.1 μg/m*^*3*^)	Ref.	Ref.	Ref.	Ref.	Ref.	Ref.	Ref.	Ref.
Group 4 (*mean 28.9 μg/m*^*3*^)	−0.055 (−0.21, 0.10)	−0.014 (−0.14, 0.11)	−0.115 (−0.27, 0.04)	−0.061 (−0.19, 0.07)	−0.056 (−0.25, 0.13)	−0.019 (−0.19, 0.15)	0.029 (−0.12, 0.18)	0.037 (−0.10, 0.17)
Group 5 (*mean 36.3 μg/m*^*3*^)	−0.267* (−0.53, −0.01)	−0.241* (−0.46, −0.02)	−0.333* (−0.62, −0.04)	−0.291* (−0.54, −0.04)	−0.369** (−0.64, −0.10)	−0.328* (−0.59, −0.07)	−0.148 (−0.39, 0.09)	−0.143 (−0.37, 0.08)
Total PM_2.5_	Model 1	Model 2	Model 1	Model 2	Model 1	Model 2	Model 1	Model 2
Mean PM_2.5_ (centered)	−0.025 (−0.06, 0.01)	−0.008 (−0.04, 0.03)	−0.027 (−0.07, 0.02)	−0.005 (−0.05, 0.04)	−0.048* (−0.09, −0.01)	−0.039* (−0.08, −0.00)	−0.016 (−0.05, 0.02)	−0.010 (−0.05, 0.03)
Group 1 (*mean 9.7μg/m*^*3*^)	−0.050 (−0.21, 0.11)	−0.065 (−0.20, 0.07)	−0.020 (−0.18, 0.15)	−0.049 (−0.19, 0.09)	0.060 (−0.11, 0.23)	0.075 (−0.07, 0.22)	−0.097 (−0.25, 0.06)	−0.103 (−0.24, 0.05)
Group 2 (*mean 11.5μg/m*^*3*^)	Ref.	Ref.	Ref.	Ref.	Ref.	Ref.	Ref.	Ref.
Group 3 (*mean 12.8μg/m*^*3*^)	0.058 (−0.06, 0.18)	0.059 (−0.05, 0.17)	0.059 (−0.06, 0.18)	0.065 (−0.04, 0.17)	0.012 (−0.14, 0.16)	0.021 (−0.12, 0.16)	0.063 (−0.06, 0.18)	0.060 (−0.05, 0.17)
Group 4 (*mean 15.2μg/m*^*3*^)	−0.460*** (−0.71, −0.21)	−0.334** (−0.55, −0.12)	−0.434** (−0.74, −0.13)	−0.292* (−0.57, −0.01)	−0.332** (−0.55, −0.11)	−0.222* (−0.44, −0.01)	−0.442*** (−0.66, −0.23)	−0.376*** (−0.58, −0.17)

*Notes*: Sources: English Longitudinal Study of Ageing (ELSA), Harmonized Cognitive Assessment Protocol (HCAP) substudy of ELSA, and Gateway to Global Aging Environmental Exposome Data for England (*N* = 1 127). For all scores, negative β indicates worse cognitive performance. Model 1 is adjusted for age and sex. Model 2 is further adjusted for age at completion of highest education qualification, wealth quintiles, urbanicity, deprivation index quintiles, and cognitive function at baseline. All covariates were drawn from ELSA Wave 4 (2008–2009). NO_2_ (µg/m^3^) = nitrogen dioxide; PM_2.5_ (µg/m^3^) = particulate matter with aerodynamic diameters less than 2.5 μm. Values in brackets represent 95% confidence intervals. *p < .05, **p < .01, ***p < .001. Weighted data.

For total PM_2.5_, when we assessed the relationship between 8-year mean exposure and cognitive performance, we only found an association with the cognitive domain related to language (β = −.039; 95% CI = [−0.08, −0.00]). However, when trajectory groups were considered, we found that those exposed to the highest levels of PM_2.5_ (at an average level of 15.16 μg/m^3^) had consistently worse cognitive scores than those who experienced an average level of exposure to PM_2.5_ (11.48 μg/m^3^) in the years under study. This relationship was observed for both overall cognitive function (β = −.334; 95% CI = [−0.55, −0.12]) as well as executive function (β = −.292; 95% CI = [−0.57, −0.01]), language (β = −.222; 95% [CI = −0.44, −0.01]), and memory (β = −.376; 95% CI = [−0.58, −0.17]) though there was no clear evidence of a compelling concentration-response function across the groups.

Tables 3 and 4 report fully adjusted associations between source-specific and fuel-specific PM_2.5_ and cognitive function (with basic Models 1 available in Supplementary Tables S5 and S6). Overall, we observed weak or no associations between source-specific PM_2.5_ and cognitive performance, with the exception that PM_2.5_ from industry and residential combustion were associated with worse language scores (β = −.364; 95% CI = [−0.64, −0.09] and β = −.321; 95% CI = [−0.54, −0.11], respectively); a similar direction and strength of associations were found when industry and residential combustion were examined using trajectory groups. Agriculture and road traffic also demonstrated similar trends though these could not be distinguished from no associations. The only cognitive domain associated with fuel-specific emissions was language where overall, lower levels of PM_2.5_ derived from solid biofuel, coal, as well as liquid oil and natural gas combustion were all associated with better performance (Table 4). Analysis using IQR change in air pollutions, that account for differences across the observed ranges in concentrations, are available in Supplementary Table S7.

**Table 3.** Results of Multiple Linear Regression for the Association Between Sector-Specific Sources of PM_2.5_ and Cognitive Performance

		Overall Cognition	Executive Function	Language	Memory
Agriculture	Mean PM_2.5_ (centered)	0.005 (−0.11, 0.12)	0.030 (−0.09, 0.15)	−0.106 (−0.22, 0.01)	0.003 (−0.12, 0.13)
	Group 1 (*mean 2.4μg/m*^*3*^)	−0.052 (−0.21, 0.11)	−0.046 (−0.21, 0.12)	0.165 (−0.02, 0.35)	−0.056 (−0.24, 0.13)
	Group 2 (*mean 2.9μg/m*^*3*^)	Ref.	Ref.	Ref.	Ref.
	Group 3 (*mean 3.4μg/m*^*3*^)	0.021 (−0.09, 0.13)	0.033 (−0.08, 0.14)	−0.037 (−0.18, 0.11)	0.059 (−0.07, 0.18)
	Group 4 (*mean 3.9μg/m*^*3*^)	−0.011 (−0.17, 0.15)	0.014 (−0.16, 0.19)	−0.045 (−0.22, 0.13)	−0.005 (−0.16, 0.15)
Industry	Mean PM_2.5_ (centered)	−0.093 (−0.35, 0.16)	−0.068 (−0.36, 0.22)	−0.364* (−0.64,−0.09)	−0.084 (−0.36, 0.19)
	Group 1 (*mean 0.5μg/m*^*3*^)	−0.020 (−0.22, 0.18)	0.016 (−0.19, 0.22)	0.095 (−0.12, 0.31)	−0.053 (−0.28, 0.17)
	Group 2 (*mean 0.8μg/m*^*3*^)	Ref.	Ref.	Ref.	Ref.
	Group 3 (*mean 1.0μg/m*^*3*^)	−0.047 (−0.06, 0.15)	0.079 (−0.03, 0.19)	−0.011 (−0.15, 0.12)	0.051 (−0.06, 0.16)
	Group 4 (*mean 1.3μg/m*^*3*^)	−0.003 (−0.14, 0.15)	−0.005 (−0.16, 0.15)	−0.151 (−0.33, 0.02)	0.020 (−0.13, 0.17)
Energy	Mean PM_2.5_ (centered)	0.009 (−0.18, 0.20)	0.087 (−0.10, 0.28)	−0.090 (−0.35, 0.17)	−0.055 (−0.27, 0.16)
	Group 1 (*mean 0.8μg/m*^*3*^)	−0.007 (−0.12, 0.11)	0.019 (−0.10, 0.14)	0.083 (−0.06, 0.23)	−0.056 (−0.18, 0.07)
	Group 2 (*mean 1.0μg/m*^*3*^)	Ref.	Ref.	Ref.	Ref.
	Group 3 (*mean 1.3μg/m*^*3*^)	−0.061 (−0.19, 0.06)	0.031 (−0.10, 0.16)	−0.117 (−0.26, 0.03)	−0.098 (−0.22, 0.03)
	Group 4 (*mean 1.6μg/m*^*3*^)	−0.028 (−0.18, 0.13)	0.021 (−0.16, 0.20)	0.084 (−0.13, 0.30)	−0.148 (−0.31, 0.01)
Residential	Mean PM_2.5_ (centered)	−0.096 (−0.30, 0.11)	−0.079 (−0.31, 0.15)	−0.321** (−0.54,−0.11)	−0.079 (−0.30, 0.14)
	Group 1 (*mean 0.7μg/m*^*3*^)	−0.131 (−0.26, 0.00)	−0.129 (−0.27, 0.01)	−0.011 (−0.15, 0.13)	−0.138 (−0.28, 0.00)
	Group 2 (*mean 1.0μg/m*^*3*^)	Ref.	Ref.	Ref.	Ref.
	Group 3 (*mean 1.2μg/m*^*3*^)	−0.073 (−0.18, 0.04)	−0.032 (−0.14, 0.08)	−0.144* (−0.29,−0.00)	−0.063 (−0.18, 0.05)
	Group 4 (*mean 1.5μg/m*^*3*^)	−0.121 (−0.27, 0.02)	−0.136 (−0.29, 0.02)	−0.210* (−0.39,−0.03)	−0.102 (−0.26, 0.05)
Road traffic	Mean PM_2.5_ (centered)	0.102 (−0.21, 0.41)	0.039 (−0.30, 0.38)	0.025 (−0.30, 0.35)	0.051 (−0.29, 0.40)
	Group 1 (*mean 0.8μg/m*^*3*^)	−0.091 (−0.21, 0.03)	−0.094 (−0.22, 0.03)	0.075 (−0.06, 0.21)	−0.114 (−0.24, 0.02)
	Group 2 (*mean 1.0μg/m*^*3*^)	Ref.	Ref.	Ref.	Ref.
	Group 3 (*mean 1.1μg/m*^*3*^)	−0.027 (−0.14, 0.09)	−0.029 (−0.15, 0.09)	−0.018 (−0.17, 0.14)	−0.036 (−0.15, 0.08)
	Group 4 (*mean 1.3μg/m*^*3*^)	−0.101 (−0.27, 0.07)	−0.156 (−0.34, 0.03)	−0.021 (−0.19, 0.15)	−0.095 (−0.27, 0.08)

*Notes*: Sources: English Longitudinal Study of Ageing (ELSA), Harmonized Cognitive Assessment Protocol (HCAP) substudy of ELSA, and Gateway to Global Aging Environmental Exposome Data for England (*N* = 1 127). For all scores, negative β indicates worse cognitive performance. All results presented in this table are adjusted for age, sex, age at completion of highest education qualification, wealth quintiles, urbanicity, deprivation index quintiles, and cognitive function at baseline (Model 2). All covariates were drawn from ELSA Wave 4 (2008–2009). Results from Model 1 (that adjusted for age and sex) are available in the Supplementary Table 5. PM_2.5_ (µg/m^3^) = particulate matter with aerodynamic diameters less than 2.5 μm. Values in brackets represent 95% confidence intervals. *p < .05, **p < .01, ***p < .001. Weighted data.

**Table 4.** Results of Multiple Linear Regression for the Association Between Fuel-Specific Sources of PM_2.5_ and Cognitive Performance

		Overall Cognition	Executive Function	Language	Memory
Biofuel	Mean PM_2.5_ (centered)	−0.058 (−0.22, 0.10)	−0.033 (−0.21, 0.14)	−0.236** (−0.41,−0.07)	−0.059 (−0.23, 0.11)
	Group 1 (*mean 0.7μg/m*^*3*^)	−0.106 (−0.29, 0.08)	−0.057 (−0.26, 0.14)	0.044 (−0.16, 0.25)	−0.140 (−0.35, 0.07)
	Group 2 (*mean 1.0μg/m*^*3*^)	Ref.	Ref.	Ref.	Ref.
	Group 3 (*mean 1.3μg/m*^*3*^)	−0.034 (−0.15, 0.08)	0.030 (−0.09, 0.15)	−0.074 (−0.21, 0.06)	−0.058 (−0.18, 0.06)
	Group 4 (*mean 1.7μg/m*^*3*^)	−0.031 (−0.16, 0.10)	0.023 (−0.11, 0.16)	−0.159* (−0.31,−0.01)	−0.059 (−0.20, 0.08)
Coal	Mean PM_2.5_ (centered)	−0.125 (−0.47, 0.22)	0.017 (−0.34, 0.37)	−0.525** (−0.91,−0.14)	−0.155 (−0.54, 0.23)
	Group 1 (*mean 0.4μg/m*^*3*^)	−0.080 (−0.27, 0.11)	−0.075 (−0.27, 0.12)	−0.012 (−0.20, 0.18)	−0.067 (−0.27, 0.14)
	Group 2 (*mean 0.6μg/m*^*3*^)	Ref.	Ref.	Ref.	Ref.
	Group 3 (*mean 0.7μg/m*^*3*^)	0.041 (−0.06, 0.15)	0.036 (−0.07, 0.15)	−0.081 (−0.22, 0.05)	0.075 (−0.04, 0.19)
	Group 4 (*mean 0.9μg/m*^*3*^)	−0.127 (−0.27, 0.01)	−0.078 (−0.23, 0.08)	−0.238** (−0.39,−0.08)	−0.103 (−0.24, 0.04)
Oil and gas	Mean PM_2.5_ (centered)	0.013 (−0.14, 0.11)	−0.019 (−0.16, 0.12)	−0.133* (−0.26,−0.00)	−0.002 (−0.15, 0.14)
	Group 1 (*mean 2.5μg/m*^*3*^)	−0.086 (−0.23, 0.06)	−0.115 (−0.26, 0.03)	0.212* (0.05, 0.37)	−0.124 (−0.29, 0.03)
	Group 2 (*mean 3.0μg/m*^*3*^)	Ref.	Ref.	Ref.	Ref.
	Group 3 (*mean 3.4μg/m*^*3*^)	0.065 (−0.04, 0.17)	0.021 (−0.09, 0.13)	0.099 (−0.04, 0.23)	0.070 (−0.04, 0.18)
	Group 4 (*mean 4.0μg/m*^*3*^)	−0.098 (−0.28, 0.09)	−0.150 (−0.35, 0.05)	−0.019 (−0.22, 0.18)	−0.099 (−0.29, 0.08)

*Notes*: Sources: English Longitudinal Study of Ageing (ELSA), Harmonized Cognitive Assessment Protocol (HCAP) substudy of ELSA, and Gateway to Global Aging Environmental Exposome Data for England (*N* = 1 127). For all scores, negative β indicates worse cognitive performance. All results presented in this table are adjusted for age, sex, age at completion of highest education qualification, wealth quintiles, urbanicity, deprivation index quintiles, and cognitive function at baseline (Model 2). All covariates were drawn from ELSA Wave 4 (2008–2009). Results from Model 1 (that adjusted for age and sex) are available in the Supplementary Table 6. PM_2.5_ (µg/m^3^) = particulate matter with aerodynamic diameters less than 2.5 μm. Values in brackets represent 95% confidence intervals. *p < .05, **p < .01, ***p < .001. Weighted data.

## Discussion

In this population-based study of English respondents aged 65 and older, air pollution exposure was associated with poorer cognitive performance, both overall and in the specific domains of executive function, language, and, to some extent, memory. Our findings are broadly in line with associations found in previous studies although the directions of associations are not always consistent. For instance, the association between exposure to the highest levels of NO_2_ and poorer cognitive performance (overall as well as executive function and language domains) was in line with Zare Sakhvidi et al. (23) but not with other studies that found links with dimensions of memory or even higher scores of cognition for higher exposure to NO_2_ (19,21,22). Similarly, exposure to the highest levels of PM_2.5_ was associated with lower cognitive scores overall and in all 3 domains of cognition studied, but these findings are not always in line with previous studies that have found associations for some but not all domains of cognition (16–22). Our study also extends the existing literature by examining associations of cognitive performance with sector- and fuel-specific PM_2.5_ emissions. We found that higher levels of PM_2.5_ from the industry and residential sectors and fuel combustion were consistently negatively associated only with the language domain. Although previous studies have mostly investigated associations between BC and cognitive performance with inconsistent findings (23–26,43), our results suggest that it is critical to consider sources of air pollution and their potential impact on different domains of cognition.

In our study, results also suggest a consistent monotonic concentration-response only in the associations between language and PM_2.5_, as well as language and sector- and fuel-specific PM_2.5_ emissions. Although the biological underpinnings of the association between pollutant exposure and poor cognitive performance are still unclear (44), it has been suggested that increased air pollution exposure is most strongly associated with impairment in the temporal lobe (45), which is essential for language and semantic fluency (46–48). However, more research is needed to disentangle the links between (source-specific) PM_2.5_ emissions and language and the potential biological mechanisms affecting cognitive domains differently. For the other air pollutants and cognitive domains, the associations are inverted j-shaped, similar to findings reported elsewhere (12,49,50). The suggestion that older people exposed to the lowest level of air pollution have lower cognitive scores than those exposed to average levels contradicts our initial hypothesis. Although our study adjusts for key individual and neighborhood-level characteristics, it is still possible that the advantages for cognition of living in an area with lower levels of pollution are offset by other factors such as economic development, access to medical insurance and health services, or housing characteristics. Future research should explore this issue with different data source(s) that allow the inclusion of a wider range of potential relevant confounders.

Some of the inconsistencies between our findings and previous studies are also likely driven by the apparent differences across study designs, settings, and analytical strategies. For example, the tests used to assess global and domain-specific cognitive scores, as well as the methods used to construct and operationalize them, were different from one study to another. Moreover, the sample characteristics themselves were quite different—as an illustration, the mean age of the study participants ranged from 57 to 76 years old, and this range might well influence the scores in the cognitive tests, the exposure to air pollution, and the selectivity of the sample. Additionally, outdoor air pollution in previous studies was collected between 2000 and 2017 with exposure ranging between 1 and 10 years prior to cognitive testing; the median values of exposures ranged from 9.9 to 25.1 μg/m^3^ for PM_2.5_ and from 25.5 to 48.1 μg/m^3^ for NO_2_ (reflecting different levels of exposure to air pollution in different geographical settings); and air pollution was operationalized as continuous but also categorical (tertiles or quartiles, or according to recommendations set by policymakers).

Even in our study, where we adopted 2 different strategies to summarize cumulative past long-term exposure to outdoor air pollution, we found differences in the magnitude and direction of these associations depending on whether we used continuous or categorical measurements. On the one hand, when we analyzed the average concentration during the period of exposure as a linear term (ie, cognitive function vs. a unit change from the mean), our results reached the conventional threshold level of 5% for statistical significance only for language, for both NO_2_ and PM_2.5_. Partly, this could be caused by power issues as the sample was relatively small, and the estimated 95% confidence intervals just overlapped zero, with the direction of associations as expected. On the other hand, when we considered these exposures as categorical groups based on their levels and trends, we found that older adults experiencing the highest levels of outdoor air pollution had consistently poorer cognitive performance than those exposed to average levels of NO_2_ and PM_2.5_, with especially compelling associations for language and PM_2.5_. Although the percentage of respondents classified in the group exposed to the highest levels of NO_2_ (at an average of 36.34 μg/m^3^) and PM_2.5_ (at an average of 15.16 μg/m^3^) is relatively small at 6%–7%, this represents more than half a million people aged 65 and older in England. Moreover, even though these groups have experienced in absolute terms the largest drop in outdoor air pollution levels, efforts to reduce air pollution concentration should continue, with a specific focus on those residential areas where outdoor pollution levels remain the highest. In our study, all respondents, even those at the lowest levels of outdoor air pollution, were exposed to air pollution concentrations above the latest 2021 Air Quality Guidelines by the World Health Organization (51).

### Strengths and Limitations

This study’s strengths include using a representative sample of the general population of older people living in private households in England and using an extensive battery of cognitive tests designed to assess key cognitive domains affected by cognitive aging, including memory, executive function, and language. Moreover, we had long-term exposure data measured up to 10 years before the cognitive performance tests administered in the ELSA-HCAP study. We were also able to link air pollution data using the point location of each exact residential address (rather than a grid around the location of residence) and account for changes in the residential address of the participants during this time, reducing potential exposure misclassification. Also, in this study, we accounted for a range of different sources of PM_2.5_, and these were isolated by removing each source individually from a chemical-transport dispersion model, leading to better specificity. The ability to investigate long-term exposure to NO_2_ and PM_2.5_, including source-specific PM_2.5_, increases our understanding of the neurotoxicity of air pollutants, even though further studies are needed to explore the role of components of particulate matter on cognitive performance. A novel aspect of this study is the use of group-based trajectory modeling to identify groups of older people exposed to different levels (and trends) of outdoor air pollution which is less common in air pollution epidemiology. In this work, we acknowledge that trajectories did not reveal changing patterns between individuals across the study period (such as increasing, decreasing, and consistently high or low levels of exposure to air pollution), and the population under study *essentially* experienced similar decreases in exposure to air pollution (though starting at different levels and with different changes over time). However, this method could be helpful to differentiate trajectories of exposure to air pollutants in other geographical settings (with more heterogeneity and contrasting changes in concentrations of air pollutants or where regulations impact air pollution across different populations (52,53)). Furthermore, by using group trajectories of pollutants, we were able to capture more heterogeneity in exposure levels than when modeling the mean exposure. Also, unlike quartiles, tertiles, or other splits of the data based on arbitrary cutoffs of the data, group-based trajectory modeling identifies homogenous groups that share similar trajectories. Other strengths of this work include the availability of detailed individual and area-level information on key factors that could confound our associations, including a measure of baseline cognition. Finally, ELSA is part of a harmonized group of studies initially developed to facilitate cross-national comparisons (with more than 40 countries around the world using the family of Health and Retirement Studies [HRS]). In this study, we used data on cognitive performance based on the HCAP (conducted in dozens of countries in the HRS-style aging surveys) and long-term exposure to outdoor air pollution that is currently being harmonized for up to 8 countries of the HRS family. Future studies should exploit this data harmonization effort by repeating these analyses in different geographical and social contexts (and possibly meta-analyzing findings). Given the current diversity of study designs, exposures, and endpoints, the availability of comparable measures will provide stronger insights into the links between outdoor air pollution exposure and cognitive aging research.

Despite these advantages, our study has some limitations. First, although air pollutant data were measured over 10 years pre-HCAP assessment, these years might not be representative of exposures over longer terms (and ideally across an individual’s entire lifetime or at different stages of the life course). Therefore, we might be unable to capture the true magnitude of the association between long-term exposure to air pollution and cognitive performance. Second, previous studies have suggested that the duration of exposure at different levels of intensity is as important as the overall intensity of exposure (49,54). In our study, however, we use yearly averages of exposures, failing to account for the duration of exposure to high NO_2_ and PM_2.5_ concentrations (measured, for instance, as the number of days or months where concentrations are above certain thresholds) and therefore to elucidate on the role of (cumulative) short-term impacts of air pollutants on cognition. Third, although our air pollution data were collected at the exact location of respondents’ residential addresses, our study is limited to a small sample of respondents living in England, which might not accurately represent the overall trends of the country. Moreover, our measures of air pollution and specific sources of air emissions are based on global models; future studies might want to investigate whether and to what extent our findings would be replicated when using larger and less selected samples as well as country-specific models to estimate air pollution and their source contributions in particular. Fourth, as with all observational studies, we cannot completely rule out the possibility that the associations we observed are attributable to unmeasured confounding. Also, although we used survey weights to minimize selection bias and nonresponse, it is probable that those who survived for the duration of the study and who agreed to participate in HCAP were a selected sample to some extent (with higher probability of response among those with higher cognitive scores). If this population was healthier and more immune to the long-term exposures to air pollution, this might underestimate the effect sizes observed. Moreover, it is worth noting that HCAP did not include respondents living in long-term care or other institutional settings and was predominantly of White European ancestry. Finally, because of the relatively small sample size, we did not investigate heterogeneities in the impact of air pollution exposure by gender, education, wealth, and other sociodemographic characteristics.

In summary, air pollution has been suggested as a modifiable risk factor for cognitive impairment. In our study, we found associations between exposure to NO_2_ and PM_2.5_ and poor cognitive performance, particularly at higher levels of concentration. Our data further indicate that key emission sources might be important particularly for the domain of language, although more research is needed to confirm these findings. Older people’s cognitive performance might benefit from continued efforts to reduce levels of exposure to air pollution, particularly where outdoor pollution levels are the highest.

## Supplementary Material

glaf060_suppl_Supplementary_Materials

## Data Availability

All ELSA data are available through the UK Data Service (SN 5050).
