# Supplementary material for: Cognitive Performance and Long-term Exposure to Outdoor Air Pollution: Findings From the Harmonized Cognitive Assessment Protocol Substudy of the English Longitudinal Study of Ageing (ELSA-HCAP)
Source: J Gerontol A Biol Sci Med Sci. 2025 Mar 17;80(5):glaf060. doi: 10.1093/gerona/glaf060 (PMC11998569; doi:10.1093/gerona/glaf060)
Supplement: glaf060_suppl_Supplementary_Materials [file glaf060_suppl_supplementary_materials.pdf]

Cognitive Performance and Long-term Exposure to Outdoor Air Pollution: Findings From the Harmonized Cognitive Assessment Protocol Substudy of the English Longitudinal Study of Ageing (ELSA-HCAP)

Supplementary Online Content

Table S1 – List of sources with descriptions of primary emissions

Figure S1 – Distribution of harmonised general and domain-specific cognitive factor scores in ELSA-HCAP

Figure S2 – Causal pathways blocked in the full-adjusted model

Table S2 – Summary measures of outdoor air pollution by year

Table S3 – Comparison of goodness of fit criteria for group-based trajectory modelling models of NO_2 concentration

Table S4 – Comparison of goodness of fit criteria for group-based trajectory modelling models of PM2.5 concentration

Figure S3 – Trajectories of sector-specific $PM_{2.5}$ and characteristics of the groups

Figure S4 – Trajectories of fuel-specific $PM_{2.5}$ and characteristics of the groups

Table S5 – Associations (95% confidence intervals) between outdoor sector-specific concentrations of $PM_{2.5}$ and cognitive performance in the ELSA-HCAP (2018)

Table S6 – Associations (95% confidence intervals) between outdoor fuel-specific concentrations of $PM_{2.5}$ and cognitive performance in the ELSA-HCAP (2018)

Table S1 – List of sources with descriptions of primary emissions

Sector	Description of primary emission	% of source
**Agriculture**	Includes manure management, soil fertilizer emissions, rice cultivation, enteric fermentation, and other agriculture	27.0
**Industry**	Industry combustion, including industrial combustion (iron and steel, non-ferrous metals, chemicals, pulp and paper, food and tobacco, non-metallic minerals, construction, transportation equipment, machinery, mining and quarrying, wood products, textile and leather, and other industry combustion); non-combustion industrial processes and product use (cement production, lime production, other minerals, chemical industry, metal production, food, beverage, wood, pulp, and paper, and other noncombustion industrial emissions); and solvents production and application (degreasing and cleaning, paint application, chemical products manufacturing and processing, and other product use)	8.0
**Energy production**	Energy production, including electricity and heat production, fuel production and transformation, oil and gas fugitive/flaring, and fossil fuel fires	9.0
**Residential**	Includes residential heating and cooking from solid biofuel combustion, coal combustion, and all non-coal non-solid biofuel residential combustion	8.6
**Road traffic**	Road transportation, including cars, motorcycles, heavy and light duty trucks and buses	8.7
**Fuel**		
**Biofuel**	Solid biofuel (or biomass) combustion	10.3
**Coal**	Hard coal, brown coal, coal coke	5.5
**Oil and Gas**	Liquid oil and natural gas combustion –light and heavy oil, diesel oil, and natural gas	26.8

Figure S1 – Distribution of harmonised general and domain-specific cognitive factor scores in ELSA-HCAP

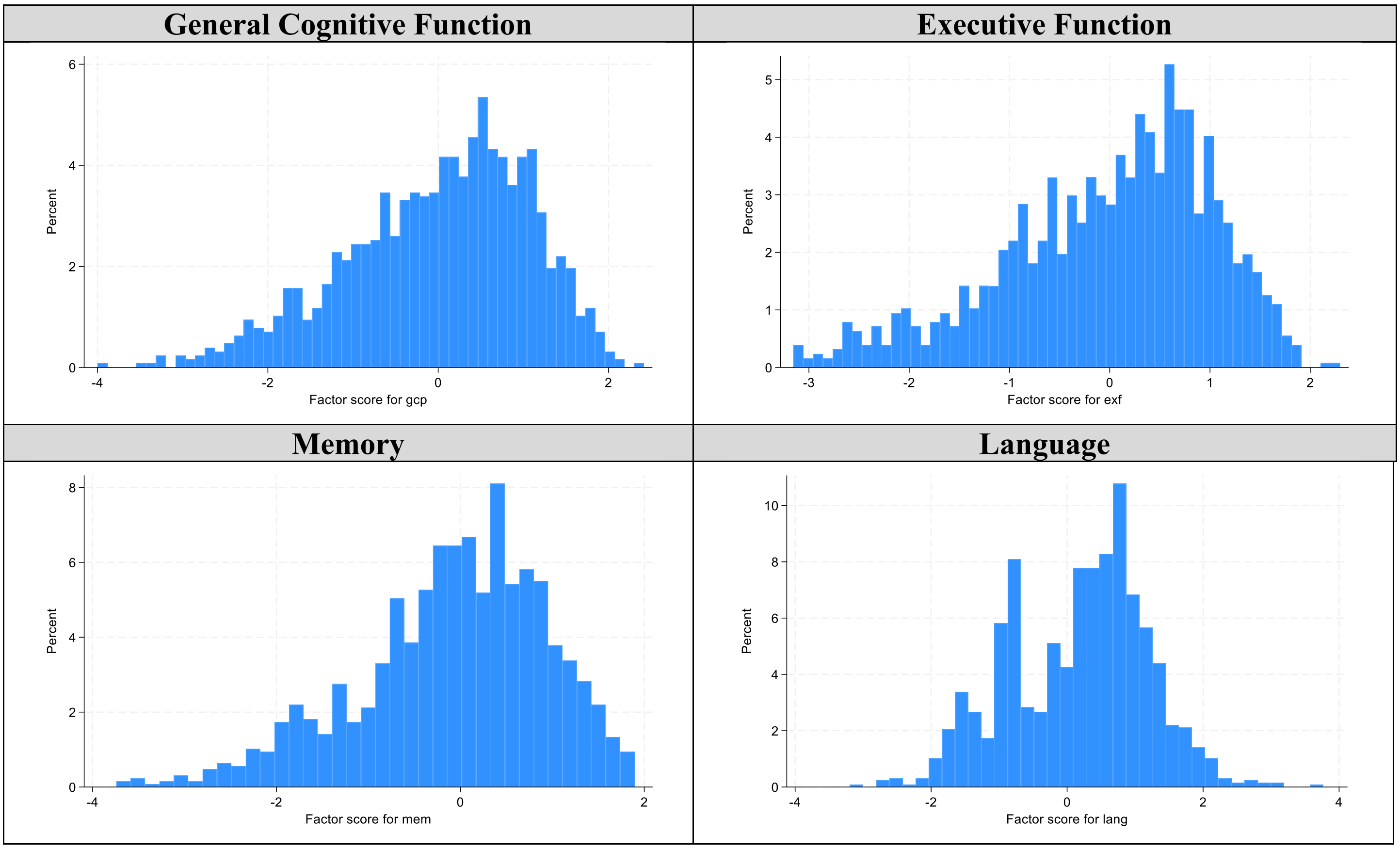

Source: Harmonised Cognitive Assessment Protocol Sub-Study of the English Longitudinal Study of Ageing (ELSA-HCAP). The histograms show general cognitive function and domain-specific cognitive factors. Bar heights in each histogram indicate the percentage of observations with a value along the x-axis.

Figure S2 – Causal pathways blocked in the full-adjusted model

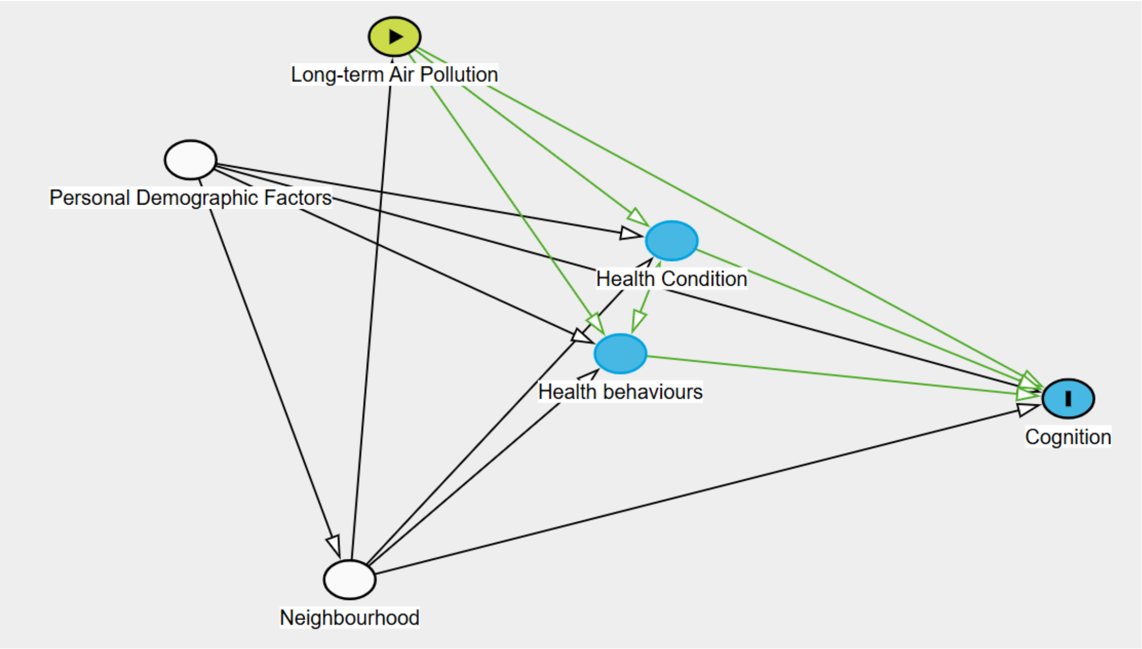

As shown in this DAG, we did not control for health conditions (such as cardiovascular, respiratory or other chronic diseases) or health behaviours (such as smoking or physical activity) as these covariates fall in the causal pathway between long-term exposure to air pollution and cognition and could underestimate the effect of long-term air pollution on cognition or introduce bias in our results.

Table S2 – Summary measures of outdoor air pollution by year

	NO_2 µg/m^3		$PM_{2.5}$ µg/m^3	
**Year**	**Mean (SD)**	**Median (IQR)**	**Mean (SD)**	**IQR**
**2008**	24.18 (6.41)	24.44 (20.68; 28.20)	n/a	n/a
**2009**	23.43 (6.37)	22.56 (18.80; 28.20)	n/a	n/a
**2010**	24.70 (6.50)	24.44 (20.68; 28.20)	13.52 (1.83)	13.64 (12.30; 14.61)
**2011**	23.81 (6.32)	24.44 (20.68; 28.20)	13.76 (1.79)	13.88 (12.54; 14.79)
**2012**	22.97 (6.56)	22.56 (18.80; 26.32)	12.44 (1.64)	12.59 (11.36; 13.41)
**2013**	22.04 (6.38)	22.56 (18.80; 26.32)	12.11 (1.57)	12.24 (11.08; 13.04)
**2014**	21.95 (6.31)	22.56 (18.80; 26.32)	11.23 (1.44)	11.32 (10.28; 12.05)
**2015**	21.70 (6.29)	20.68 (16.92; 26.32)	11.34 (1.41)	11.44 (10.41; 12.12)
**2016**	22.77 (6.41)	22.56 (18.80; 26.32)	10.43 (1.33)	10.54 (9.58; 11.18)
**2017**	21.36 (6.37)	20.68 (16.92; 26.32)	10.33 (1.28)	10.44 (9.48; 11.11)
**Mean**	22.89 (6.36)	22.74 (18.80; 27.07)	11.89 (1.53)	12.01 (10.87; 12.78)

Sources: Gateway to Global Aging Environmental Exposome Data for England. The sample is restricted to participants of the Harmonised Cognitive Assessment Protocol Sub-Study of the English Longitudinal Study of Ageing (ELSA-HCAP, N=1,127). NO_2 (µg/m^3) = Nitrogen dioxide; $PM_{2.5}$ (µg/m^3) = Particulate matter with aerodynamic diameters less than 2.5 µm. SD=Standard Deviation; IQR=Inter Quartile Range

Table S3 – Comparison of goodness of fit criteria for group-based trajectory modelling models of NO_2 concentration

N classes	AIC	BIC	cBIC	Entropy	Class membership
1	-38417.6	-38425.2	-38428.6	1	
2	-33481.0	-33503.1	-33496.2	0.954	55.8%; 44.2%
3	-30364.7	-30397.9	-30387.5	0.966	23.6%; 48.3%; 28.1%
4	-27639.5	-27683.7	-27639.5	0.976	18.6%; 38.3%; 31.3%; 11.8%
**5**	**-25898.5**	**-25953.8**	**-25936.5**	**0.973**	**17.2%, 28.4%, 26.2%, 21.8%, 6.4%**
6	-24966.2	-25032.6	-25011.8	0.963	11.9%; 13.4%; 25.3%; 23.6%; 19.3%; 6.4%
7	-25891.2	-25969.4	-25945.2	0.974	6.1%; 12.4%; 21.9%; 23.2%; 19.9%; 11.6%; 4.9%

Sources: Gateway to Global Aging Environmental Exposome Data for England (2008-2017). The sample is restricted to participants of the Harmonised Cognitive Assessment Protocol Sub-Study of the English Longitudinal Study of Ageing (ELSA-HCAP, N=1,127). Notes: NO_2=nitrogen dioxide; AIC=Akaike Information Criterion; BIC=Bayesian Information Criterion; c-BIC=sample size corrected BIC

Table S4 – Comparison of goodness of fit criteria for group-based trajectory modelling models of PM2.5 concentration

N classes	AIC	BIC	cBIC	Entropy	Class membership
1	-17579.4	-17590.2	-17587.1	1	
2	-14296.2	-14317.6	-14311.4	0.937	40.4%; 59.6%
3	-12029.8	-12061.9	-12052.6	0.971	27.8%; 61.51%; 10.7%
**4**	**-9537.4**	**-9637.5**	**-9608.4**	**0.960**	**20.4%; 33.8%; 39.2%; 6.6%**
5	-9348.9	-9402.5	-9386.9	0.980	5.2%, 20.3%, 31.8%, 36.2%, 6.5%
6	-8620.9	-8685.2	-8666.5	0.969	3.4%; 17.7%; 23.0%; 17.8%; 32.2%; 5.9%
7	-8042.5	-8117.5	-8095.7	0.978	3.3%; 13.2%; 14.5%; 23.9%; 29.1%; 11.6%; 4.4%

Sources: Gateway to Global Aging Environmental Exposome Data for England (2010-2017). The sample is restricted to participants of the Harmonised Cognitive Assessment Protocol Sub-Study of the English Longitudinal Study of Ageing (ELSA-HCAP, N=1,127). Notes: $PM_{2.5}$=particulate matter with aerodynamic diameters less than 2.5 μm; AIC=Akaike Information Criterion; BIC=Bayesian Information Criterion; c-BIC=sample size corrected BIC

Figure S3 – Trajectories of sector-specific $PM_{2.5}$ and characteristics of the groups

Agriculture

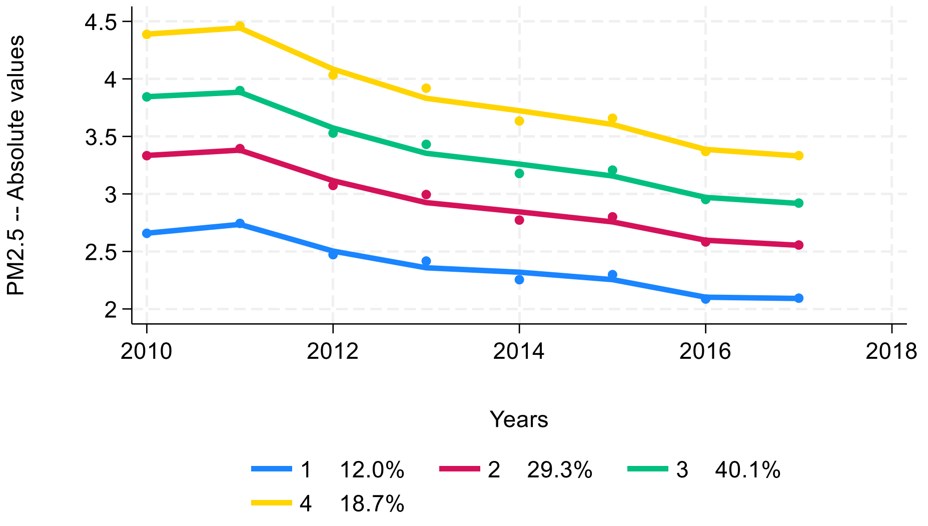

	%	Mean $PM_{2.5}$ 2010 (SD)	Mean $PM_{2.5}$ 2017 (SD)	8-year $PM_{2.5}$ mean (SD)
Group 1	12.0	2.66 (0.23)	2.09 (0.16)	2.38 (0.19)
Group 2	29.3	3.33 (0.16)	2.56 (0.11)	2.94 (0.13)
Group 3	40.1	3.84 (0.15)	2.92 (0.11)	3.37 (0.12)
Group 4	18.7	4.39 (0.29)	3.34 (0.19)	3.85 (0.23)

Industry

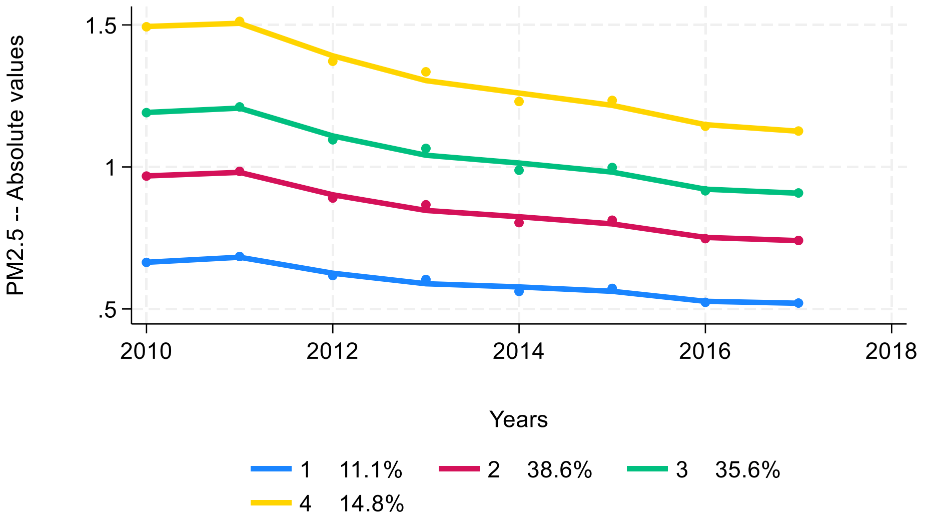

	%	Mean $PM_{2.5}$ 2010 (SD)	Mean $PM_{2.5}$ 2017 (SD)	8-year $PM_{2.5}$ mean (SD)
Group 1	11.1	0.66 (0.12)	0.52 (0.09)	0.59 (0.10)
Group 2	38.6	0.97 (0.08)	0.74 (0.05)	0.85 (0.06)
Group 3	35.6	1.19 (0.07)	0.91 (0.05)	1.05 (0.06)
Group 4	14.8	1.49 (0.12)	1.13 (0.08)	1.31 (0.10)

Energy Production

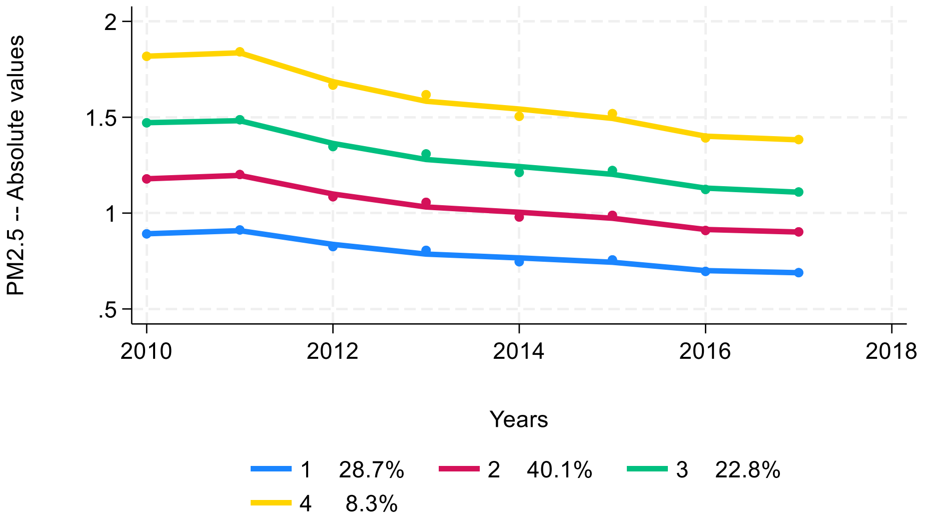

	%	Mean $PM_{2.5}$ 2010 (SD)	Mean $PM_{2.5}$ 2017 (SD)	8-year $PM_{2.5}$ mean (SD)
Group 1	28.7	0.89 (0.12)	0.69 (0.09)	0.79 (0.10)
Group 2	40.1	1.18 (0.08)	0.90 (0.06)	1.04 (0.07)
Group 3	22.8	1.47 (0.10)	1.11 (0.06)	1.28 (0.08)
Group 4	8.3	1.82 (0.20)	1.38 (0.15)	1.59 (0.17)

Residential

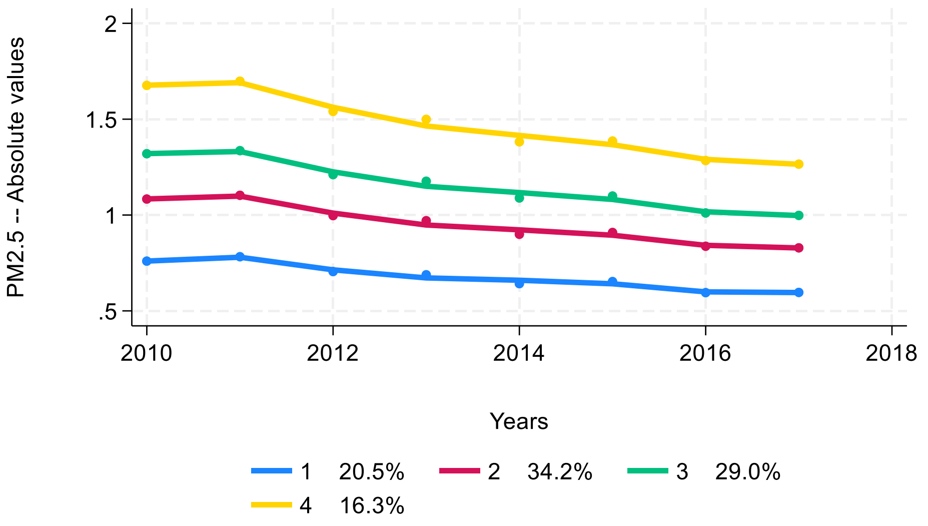

	%	Mean $PM_{2.5}$ 2010 (SD)	Mean $PM_{2.5}$ 2017 (SD)	8-year $PM_{2.5}$ mean (SD)
Group 1	20.5	0.76 (0.13)	0.60 (0.09)	0.68 (0.11)
Group 2	34.2	1.08 (0.08)	0.83 (0.06)	0.95 (0.07)
Group 3	29.0	1.32 (0.08)	1.00 (0.06)	1.16 (0.06)
Group 4	16.3	1.68 (0.14)	1.27 (0.10)	1.47 (0.12)

Road Traffic

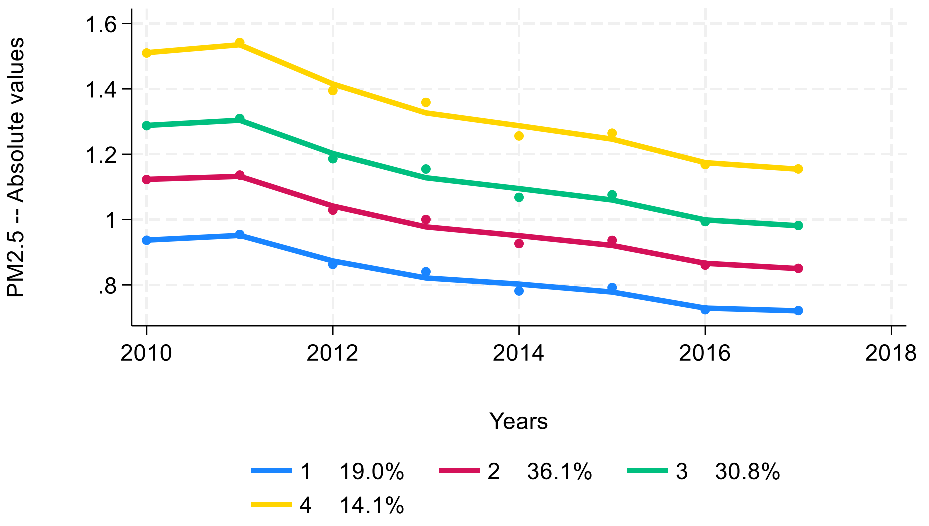

	%	Mean $PM_{2.5}$ 2010 (SD)	Mean $PM_{2.5}$ 2017 (SD)	8-year $PM_{2.5}$ mean (SD)
Group 1	19.0	0.94 (0.08)	0.72 (0.05)	0.83 (0.06)
Group 2	36.1	1.12 (0.05)	0.85 (0.04)	0.98 (0.04)
Group 3	30.8	1.29 (0.06)	0.98 (0.04)	1.13 (0.05)
Group 4	14.1	1.51 (0.09)	1.16 (0.08)	1.33 (0.08)

Sources: Gateway to Global Aging Environmental Exposome Data for England. The sample is restricted to participants of the Harmonised Cognitive Assessment Protocol Sub-Study of the English Longitudinal Study of Ageing (ELSA-HCAP, N=1,127).
$PM_{2.5}$ ($\mu g/m^3$) = Particulate matter with aerodynamic diameters less than 2.5 μm. SD=Standard Deviation.

Figure S4 – Trajectories of fuel-specific $PM_{2.5}$ and characteristics of the groups

Biofuel

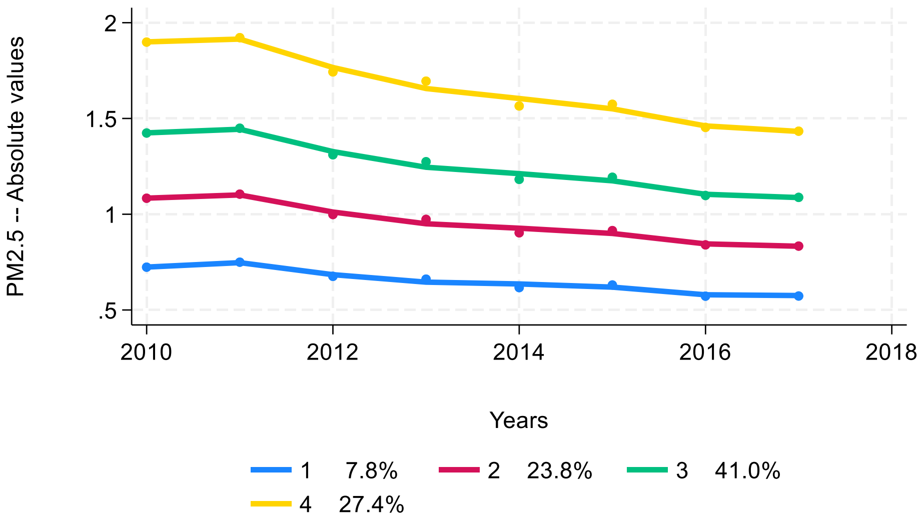

	%	Mean $PM_{2.5}$ 2010 (SD)	Mean $PM_{2.5}$ 2017 (SD)	8-year $PM_{2.5}$ mean (SD)
Group 1	7.8	0.72 (0.11)	0.57 (0.09)	0.65 (0.10)
Group 2	23.8	1.08 (0.10)	0.83 (0.07)	0.96 (0.08)
Group 3	41.0	1.42 (0.11)	1.09 (0.08)	1.25 (0.10)
Group 4	27.4	1.90 (0.18)	1.43 (0.12)	1.66 (0.15)

Coal

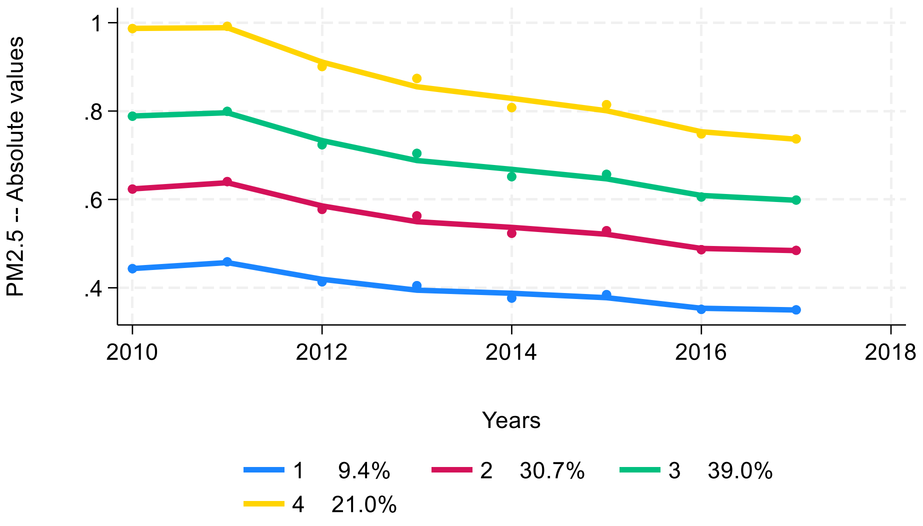

	%	Mean $PM_{2.5}$ 2010 (SD)	Mean $PM_{2.5}$ 2017 (SD)	8-year $PM_{2.5}$ mean (SD)
Group 1	9.3	0.44 (0.08)	0.35 (0.06)	0.40 (0.07)
Group 2	30.7	0.62 (0.05)	0.49 (0.04)	0.55 (0.04)
Group 3	39.0	0.79 (0.05)	0.60 (0.03)	0.69 (0.04)
Group 4	21.0	0.99 (0.07)	0.74 (0.05)	0.86 (0.06)

Oil and Gas

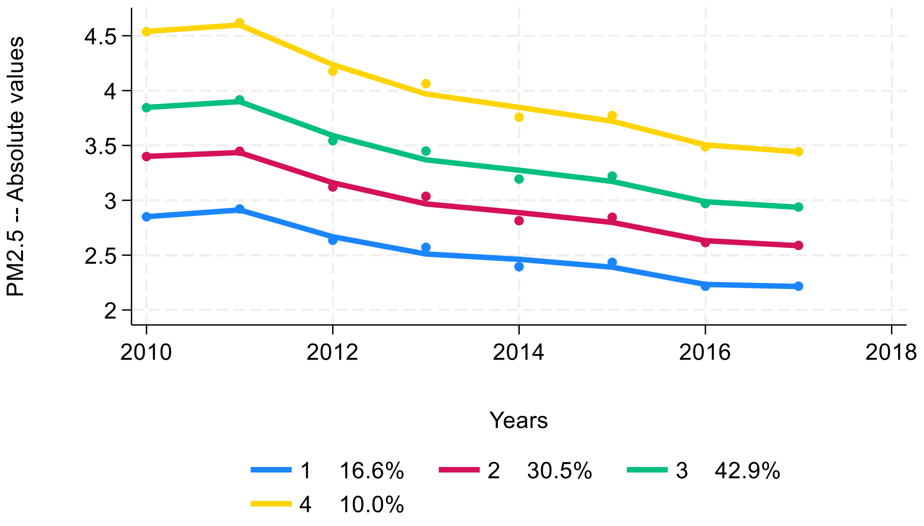

	%	Mean $PM_{2.5}$ 2010 (SD)	Mean $PM_{2.5}$ 2017 (SD)	8-year $PM_{2.5}$ mean (SD)
Group 1	16.6	2.85 (0.22)	2.22 (0.16)	2.53 (0.18)
Group 2	30.5	3.40 (0.15)	2.59 (0.10)	2.98 (0.12)
Group 3	42.9	3.84 (0.15)	2.94 (0.13)	3.38 (0.14)
Group 4	10.0	4.54 (0.31)	3.44 (0.19)	3.98 (0.24)

Sources: Gateway to Global Aging Environmental Exposome Data for England. The sample is restricted to participants of the Harmonised Cognitive Assessment Protocol Sub-Study of the English Longitudinal Study of Ageing (ELSA-HCAP, N=1,127). $PM_{2.5}$ ($\mu g/m^3$) = Particulate matter with aerodynamic diameters less than 2.5 μm. SD=Standard Deviation

Table S5 – Associations (95% confidence intervals) between outdoor sector-specific concentrations of $PM_{2.5}$ and cognitive performance in the ELSA-HCAP (2018)

		Overall Cognition		Executive Function		Language		Memory	
		**Model 1**	**Model 2**	**Model 1**	**Model 2**	**Model 1**	**Model 2**	**Model 1**	**Model 2**
**Agriculture**	**Mean $PM_{2.5}$ (centred)**	-0.063 (-0.19,0.06)	0.005 (-0.11,0.12)	-0.052 (-0.19,0.08)	0.030 (-0.09,0.15)	-0.147* (-0.27,-0.02)	-0.106 (-0.22,0.01)	-0.033 (-0.15,0.09)	0.003 (-0.12,0.13)
	**Group 1 (*mean 2.4 µg/m^3*)**	-0.101 (-0.30,0.10)	-0.052 (-0.21,0.11)	-0.084 (-0.29,0.12	-0.046 (-0.21,0.12)	0.114 (-0.10,0.33)	0.165 (-0.02,0.35)	-0.086 (-0.29,0.11)	-0.056 (-0.24,0.13)
	**Group 2 (*mean 2.9 µg/m^3*)**	Ref	Ref	Ref	Ref	Ref	Ref	Ref	Ref
	**Group 3 (*mean 3.4 µg/m^3*)**	-0.032 (-0.16,0.10)	0.021 (-0.09,0.13)	-0.016 (-0.15,0.11)	0.033 (-0.08,0.14)	-0.077 (-0.23,0.08)	-0.037 (-0.18,0.11)	0.021 (-0.11,0.15)	0.059 (-0.07,0.18)
	**Group 4 (*mean 3.8 µg/m^3*)**	-0.172 (-0.34,0.00)	-0.011 (-0.17,0.15)	-0.156 (-0.35,0.04)	0.014 (-0.16,0.19)	-0.167 (-0.35,0.02)	-0.045 (-0.22,0.13)	-0.107 (-0.26,0.05)	-0.005 (-0.16,0.15)
**Industry**	**Mean $PM_{2.5}$ (centred)**	-0.133 (-0.42,0.15)	-0.093 (-0.35,0.16)	-0.145 (-0.47,0.18)	-0.068 (-0.36,0.22	-0.372* (-0.66,-0.08)	-0.364* (-0.64,-0.09)	-0.083 (-0.35,0.19)	-0.084 (-0.36,0.19)
	**Group 1 (*mean 0.5 µg/m^3*)**	-0.042 (-0.29,0.20)	-0.020 -0.22,0.18)	0.009 (-0.24,0.26)	0.016 (-0.19,0.22)	0.035 (-0.22,0.29)	0.095 (-0.12,0.31)	-0.067 (-0.31,0.18)	-0.053 (-0.28,0.17)
	**Group 2 (*mean 0.8 µg/m^3*)**	Ref	Ref	Ref	Ref	Ref	Ref	Ref	Ref
	**Group 3 (*mean 1.0 µg/m^3*)**	0.010 (-0.11,0.13)	-0.047 (-0.06,0.15)	0.038 (-0.09,0.17)	0.079 (-0.03,0.19)	-0.037 (-0.18,0.11)	-0.011 (-0.15,0.12)	-0.034 (-0.08,0.15)	0.051 (-0.06,0.16)
	**Group 4 (*mean 1.3 µg/m^3*)**	-0.034 (-0.20,0.13)	-0.003 (-0.14,0.15)	-0.049 (-0.23,0.13)	-0.005 (-0.16,0.15)	-0.190* (-0.38,-0.00)	-0.151 (-0.33,0.02)	0.001 (-0.16,0.16)	0.020 (-0.13,0.17)
**Energy**	**Mean $PM_{2.5}$ (centred)**	-0.196 (-0.42,0.03)	0.009 (-0.18,0.20)	-0.130 (-0.36,0.10)	0.087 (-0.10,0.28)	-0.239 (-0.53,0.05)	-0.090 (-0.35,0.17)	-0.171 (-0.38,0.04)	-0.055 (-0.27,0.16)
	**Group 1 (*mean 0.8 µg/m^3*)**	0.056 (-0.08,0.19)	-0.007 (-0.12,0.11)	0.087 (-0.05,0.22)	0.019 (-0.10,0.14)	0.134 (-0.02,0.28)	0.083 (-0.06,0.23)	-0.024 (-0.15,0.10)	-0.056 (-0.18,0.07)
	**Group 2 (*mean 1.0 µg/m^3*)**	Ref	Ref	Ref	Ref	Ref	Ref	Ref	Ref
	**Group 3 (*mean 1.3 µg/m^3*)**	-0.155* (-0.30,-0.01)	-0.061 (-0.19,0.06)	-0.031 (-0.21,0.09)	0.031 (-0.10,0.16)	-0.183* (-0.34,-0.03)	-0.117 (-0.26,0.03)	-0.163* (-0.30,-0.03)	-0.098 (-0.22,0.03)
	**Group 4 (*mean 1.6 µg/m^3*)**	-0.146 (-0.34,0.04)	-0.028 (-0.18,0.13)	-0.094 (-0.31,0.11)	0.021 (-0.16,0.20)	-0.013 (-0.27,0.24)	0.084 (-0.13,0.30)	-0.221* (-0.40,-0.05)	-0.148 (-0.31,0.01)
**Residential**	**Mean $PM_{2.5}$ (centred)**	-0.090 (-0.32,0.14)	-0.096 (-0.30,0.11)	-0.102 (-0.36,0.16)	-0.079 (-0.31,0.15)	-0.301* (-0.53,-0.07)	-0.321** (-0.54,-0.11)	-0.054 (-0.27,0.17)	-0.078 (-0.30,0.14)
	**Group 1 (*mean 0.7 µg/m^3*)**	-0.117 (-0.27,0.04)	-0.131 (-0.26,0.00)	-0.102 (-0.26,0.06)	-0.129 (-0.27,0.01)	-0.033 (-0.20,0.13)	-0.011 (-0.15,0.13)	-0.134 (-0.28,0.02)	-0.138 (-0.28,0.00)
	**Group 2 (*mean 1.0 µg/m^3*)**	Ref	Ref	Ref	Ref	Ref	Ref	Ref	Ref
	**Group 3 (*mean 1.2 µg/m^3*)**	-0.030 (-0.16,0.10)	-0.073 (-0.18,0.04)	0.010 (-0.12,0.14)	-0.032 (-0.14,0.08)	-0.116 (-0.28,0.04)	-0.144* (-0.29,-0.00)	-0.030 (-0.15,0.09)	-0.063 (-0.18,0.05)
	**Group 4 (*mean 1.5 µg/m^3*)**	-0.089 (-0.26,0.08)	-0.121 (-0.27,0.02)	-0.111 (-0.29,0.07)	-0.136 (-0.29,0.02)	-0.203* (-0.39,-0.02)	-0.210* (-0.39,-0.03)	-0.075 (-0.24,0.09)	-0.102 (-0.26,0.05)
**Road Traffic**	**Mean $PM_{2.5}$ (centred)**	0.061 (-0.30,0.42)	0.102 (-0.21,0.41)	-0.039 (-0.45,0.37)	0.039 (-0.30,0.38)	0.002 (-0.37,0.37)	0.025 (-0.30,0.35)	0.054 (-0.29,0.40)	0.051 (-0.29,0.40)
	**Group 1 (*mean 0.8 µg/m^3*)**	0.011 (-0.14,0.16)	-0.091 (-0.21,0.03)	0.026 (-0.13,0.18)	-0.094 (-0.22,0.03)	0.132 (-0.03,0.29)	0.075 (-0.06,0.21)	-0.077 (-0.21,0.06)	-0.114 (-0.24,0.02)
	**Group 2 (*mean 1.0 µg/m^3*)**	Ref	Ref	Ref	Ref	Ref	Ref	Ref	Ref
	**Group 3 (*mean 1.1 µg/m^3*)**	0.042 (-0.09,0.17)	-0.027 (-0.14,0.09)	0.040 (-0.09,0.17)	-0.029 (-0.15,0.09)	0.023 (-0.14,0.19)	-0.018 (-0.17,0.14)	0.007 (-0.12,0.13)	-0.036 (-0.15,0.08)
	**Group 4 (*mean 1.3 µg/m^3*)**	-0.055 (-0.25,0.14)	-0.101 (-0.27,0.07)	-0.117 (-0.34,0.10)	-0.156 (-0.34,0.03)	0.001 (-0.19,0.19)	-0.021 (-0.19,0.15)	-0.057 (-0.24,0.13)	-0.095 (-0.27,0.08)

Sources – English Longitudinal Study of Ageing (ELSA), Harmonised Cognitive Assessment Protocol (HCAP) Sub-Study of ELSA, and Gateway to Global Aging Environmental Exposome Data for England (N=1,127). Notes: For all scores, negative β indicates worse cognitive performance. Model 1 is adjusted for age and sex. Model 2 is further adjusted for age at completion of highest education qualification, wealth quintiles, urbanicity, deprivation index quintiles, and cognitive function at baseline. All covariates were drawn from ELSA Wave 4 (2008-09). $PM_{2.5}$ (µg/m^3) =particulate matter with aerodynamic diameters less than 2.5 µm. Values in brackets represent 95% confidence intervals. * $p < 0.05$, ** $p < 0.01$, *** $p < 0.001$. Weighted data

Table S6 – Associations (95% confidence intervals) between outdoor fuel-specific concentrations of $PM_{2.5}$ and cognitive performance in the ELSA-HCAP (2018)

		Overall Cognition		Executive Function		Language		Memory	
		**Model 1**	**Model 2**	**Model 1**	**Model 2**	**Model 1**	**Model 2**	**Model 1**	**Model 2**
**Biofuel**	**Mean $PM_{2.5}$ (centred)**	-0.073<br>(-0.26,0.11)	-0.058<br>(-0.22,0.10)	-0.067<br>(-0.27,0.14)	-0.033<br>(-0.21,0.14)	-0.236*<br>(-0.42,-0.05)	-0.236**<br>(-0.41,-0.07)	-0.055<br>(-0.23,0.12)	-0.059<br>(-0.23,0.11)
	**Group 1 (*mean 0.7 μg/m^3*)**	-0.136<br>(-0.38,0.11)	-0.106<br>(-0.29,0.08)	-0.077<br>(-0.34,0.18)	-0.057<br>(-0.26,0.14)	0.012<br>(-0.26,0.24)	0.044<br>(-0.16,0.25)	-0.159<br>(-0.39,0.08)	-0.140<br>(-0.35,0.07)
	**Group 2 (*mean 1.0 μg/m^3*)**	Ref	Ref	Ref	Ref	Ref	Ref	Ref	Ref
	**Group 3 (*mean 1.3 μg/m^3*)**	-0.063<br>(-0.20,0.07)	-0.034<br>(-0.15,0.08)	0.010<br>(-0.15,0.13)	0.030<br>(-0.09,0.15)	-0.094<br>(-0.27,0.24)	-0.074<br>(-0.21,0.06)	-0.061<br>(-0.19,0.07)	-0.058<br>(-0.18,0.06)
	**Group 4 (*mean 1.7 μg/m^3*)**	-0.068<br>(-0.22,0.09)	-0.031<br>(-0.16,0.10)	-0.026<br>(-0.19,0.13)	0.023<br>(-0.11,0.16)	-0.188*<br>(-0.36,-0.02)	-0.159*<br>(-0.31,-0.01)	-0.073<br>(-0.22,0.08)	-0.059<br>(-0.20,0.08)
**Coal**	**Mean $PM_{2.5}$ (centred)**	-0.401*<br>(-0.80,-0.00)	-0.126<br>(-0.47,0.22)	-0.302<br>(-0.73,0.13)	0.017<br>(-0.34,0.37)	-0.688***<br>(-1.11,-0.26)	-0.525**<br>(-0.91,-0.14)	-0.294<br>(-0.67,0.08)	-0.155<br>(-0.54,0.23)
	**Group 1 (*mean 0.4 μg/m^3*)**	-0.150<br>(-0.38,0.08)	-0.080<br>(-0.27,0.11)	-0.126<br>(-0.36,0.10)	-0.075<br>(-0.27,0.12)	-0.092<br>(-0.32,0.13)	-0.012<br>(-0.20,0.18)	-0.121<br>(-0.34,0.10)	-0.067<br>(-0.27,0.14)
	**Group 2 (*mean 0.6 μg/m^3*)**	Ref	Ref	Ref	Ref	Ref	Ref	Ref	Ref
	**Group 3 (*mean 0.7 μg/m^3*)**	-0.005<br>(-0.13,0.12)	0.041<br>(-0.06,0.15)	-0.009<br>(-0.14,0.12)	0.036<br>(-0.07,0.15)	-0.122<br>(-0.27,0.03)	-0.081<br>(-0.22,0.05)	0.048<br>(-0.07,0.17)	0.075<br>(-0.04,0.19)
	**Group 4 (*mean 0.9 μg/m^3*)**	-0.263**<br>(-0.43,-0.10)	-0.127<br>(-0.27,0.01)	-0.222*<br>(-0.40,-0.04)	-0.078<br>(-0.23,0.08)	-0.332***<br>(-0.51,-0.15)	-0.238**<br>(-0.39,-0.08)	-0.180*<br>(-0.33,-0.03)	-0.103<br>(-0.24,0.04)
**Oil and Gas**	**Mean $PM_{2.5}$ (centred)**	-0.020<br>(-0.16,0.12)	-0.013<br>(-0.14,0.11)	-0.045<br>(-0.21,0.12)	-0.019<br>(-0.16,0.12)	-0.127<br>(-0.26,0.01)	-0.133*<br>(-0.26,-0.00)	0.004<br>(-0.13,0.14)	-0.002<br>(-0.15,0.14)
	**Group 1 (*mean 2.5 μg/m^3*)**	-0.125<br>(-0.30,0.05)	-0.086<br>(-0.23,0.06)	-0.153<br>(-0.33,0.02)	-0.115<br>(-0.26,0.03)	0.145<br>(-0.05,0.34)	0.212*<br>(0.05,0.37)	-0.144<br>(-0.32,0.03)	-0.124<br>(-0.29,0.03)
	**Group 2 (*mean 3.0 μg/m^3*)**	Ref	Ref	Ref	Ref	Ref	Ref	Ref	Ref
	**Group 3 (*mean 3.4 μg/m^3*)**	0.084<br>(-0.04,0.21)	0.065<br>(-0.04,0.17)	0.032<br>(-0.09,0.16)	0.021<br>(-0.09,0.13)	0.105<br>(-0.04,0.25)	0.099<br>(-0.04,0.23)	0.086<br>(-0.03,0.20)	0.070<br>(-0.04,0.18)
	**Group 4 (*mean 4.0 μg/m^3*)**	-0.156<br>(-0.37,0.06)	-0.098<br>(-0.28,0.09)	-0.228<br>(-0.47,0.01)	-0.150<br>(-0.35,0.05)	-0.089<br>(-0.30,0.12)	-0.019<br>(-0.22,0.18)	-0.118<br>(-0.31,0.08)	-0.099<br>(-0.29,0.08)

Sources – English Longitudinal Study of Ageing (ELSA), Harmonised Cognitive Assessment Protocol (HCAP) Sub-Study of ELSA, and Gateway to Global Aging Environmental Exposome Data for England (N=1,127). Notes: For all scores, negative β indicates worse cognitive performance. Model 1 is adjusted for age and sex. Model 2 is further adjusted for age at completion of highest education qualification, wealth quintiles, urbanicity, deprivation index quintiles, and cognitive function at baseline. All covariates were drawn from ELSA Wave 4 (2008-09). $PM_{2.5}$ (μg/m^3) = Particulate matter with aerodynamic diameters less than 2.5 μm. Values in brackets represent 95% confidence intervals. * $p < 0.05$, ** $p < 0.01$, *** $p < 0.001$. Weighted data.

Table S7 – Associations (95% confidence intervals) between outdoor air pollution concentrations of NO_2 and total $PM_{2.5}$ (using interquartile range) and cognitive performance in the ELSA-HCAP (2018)

	Overall Cognition		Executive Function		Language		Memory	
	**Model 1**	**Model 2**	**Model 1**	**Model 2**	**Model 1**	**Model 2**	**Model 1**	**Model 2**
**NO_2**	-0.105** (-0.18,-0.03)	-0.062 (-0.15,0.02)	-0.135** (-0.22,-0.05)	-0.086 (-0.18,0.01)	-0.130** (-0.21,-0.05)	-0.109* (-0.20,-0.02)	-0.045 (-0.12,0.03)	-0.020 (-0.10,0.06)
**$PM_{2.5}$**	-0.048 (-0.12,0.03)	-0.016 (-0.08,0.05)	-0.052 (-0.14,0.03)	-0.009 (-0.09,0.07)	-0.092* (-0.17,-0.02)	-0.074* (-0.14,-0.00)	-0.030 (-0.10,0.04)	-0.019 (-0.10,0.06)
**Sector-specific $PM_{2.5}$**								
**Agriculture**	-0.038 (-0.11,0.04)	0.003 (-0.07,0.007)	-0.031 (-0.11,0.05)	0.018 (-0.05,0.09)	-0.089* (-0.17,-0.01)	-0.064 (-0.14,0.01)	-0.020 (-0.09,0.05)	0.002 (-0.08,0.08)
**Industry**	-0.034 (-0.11,0.04)	-0.024 (-0.09,0.04)	-0.037 (-0.12,0.05)	-0.017 (-0.09,0.06)	-0.095* (-0.17,-0.02)	-0.093* (-0.16,-0.02)	-0.021 (-0.09,0.05)	-0.021 (-0.09,-0.05)
**Energy**	-0.065 (-0.14;0.01)	0.003 (-0.06,0.07)	-0.043 (-0.12,0.03)	0.029 (-0.03,0.09)	-0.080 (-0.17,0.2)	-0.030 (-0.12,0.06)	-0.057 (-0.13,0.01)	-0.018 (-0.09,0.05)
**Residential**	-0.029 (-0.10,0.05)	-0.031 (-0.10,0.03)	-0.032 (-0.11,0.05)	-0.025 (-0.10,0.05)	-0.096* (-0.17,-0.02)	-0.102** (-0.17,-0.03)	-0.017 (-0.08,0.05)	-0.025 (-0.09,0.05)
**Road**	0.013 (-0.07,0.09)	0.022 (-0.05,0.09)	-0.008 (-0.10,0.08)	0.009 (-0.07,0.08)	0.000 (-0.08,0.08)	0.005 (-0.07,0.08)	0.012 (-0.06,0.08)	0.011 (-0.06,0.09)
**Fuel-specific $PM_{2.5}$**								
**Biofuel**	-0.034 (-0.12,0.05)	-0.027 (-0.10,0.04)	-0.031 (-0.13,0.06)	-0.015 (-0.10,0.07)	-0.111* (-0.20,-0.02)	-0.111** (-0.19,-0.03)	-0.055 (-0.23,0.12)	-0.028 (-0.11,0.05)
**Coal**	-0.081* (-0.16,-0.00)	-0.025 (-0.09,0.04)	-0.061 (-0.15,0.03)	0.003 (-0.07,0.08)	-0.139*** (-0.22,-0.05)	-0.106** (-0.18,0.03)	-0.059 (-0.14,0.02)	-0.031 (-0.11,0.05)
**Oil and Gas**	-0.011 (-0.09,0.06)	-0.007 (-0.07,0.06)	-0.024 (-0.11,0.06)	-0.010 (-0.08,0.06)	-0.067 (-0.14,0.01)	-0.071* (-0.14,-0.00)	0.002 (-0.07,0.08)	-0.001 (-0.08,0.08)

Sources – English Longitudinal Study of Ageing (ELSA), Harmonised Cognitive Assessment Protocol (HCAP) Sub-Study of ELSA, and Gateway to Global Aging Environmental Exposome Data for England (N=1,127). Notes: For all scores, negative β indicates worse cognitive performance per **interquartile range (IQR) increase.** Model 1 is adjusted for age and sex. Model 2 is further adjusted for age at completion of highest education qualification, wealth quintiles, urbanicity, deprivation index quintiles, and cognitive function at baseline. All covariates were drawn from ELSA Wave 4 (2008-09). NO_2 ($\mu g/m^3$) = Nitrogen dioxide; $PM_{2.5}$ ($\mu g/m^3$) = Particulate matter with aerodynamic diameters less than 2.5 μm. Values in brackets represent 95% confidence intervals. * $p < 0.05$, ** $p < 0.01$, *** $p < 0.001$. Weighted data.
